# Deep Learning-Based Assessment of Brainstem Volume Changes in Spinocerebellar Ataxia Type 2 (SCA2): A Study on Patients and Preclinical Subjects

**DOI:** 10.3390/s25196009

**Published:** 2025-09-29

**Authors:** Robin Cabeza-Ruiz, Luis Velázquez-Pérez, Evelio González-Dalmau, Alejandro Linares-Barranco, Roberto Pérez-Rodríguez

**Affiliations:** 1 Center for CAD/CAM Studies, University of Holguín, Holguín 80100, Cuba; roberto.perez@uho.edu.cu; 2 Cuban Academy of Sciences, Havana 10200, Cuba; velazq63@gmail.com; 3 Centre for the Research and Rehabilitation of Hereditary Ataxias, Holguín 80100, Cuba; 4 Cuban Neuroscience Center, Havana 10200, Cuba; eglezdm02@gmail.com; 5 Robotics and Technology of Computers Lab, University of Seville, 41012 Seville, Spain; alinares@us.es; 6 Escuela Politécnica Superior (EPS), University of Seville, 41011 Seville, Spain; 7 Smart Computer Systems Research and Engineering Lab (SCORE), Research Institute of Computer Engineering (I3US), University of Seville, 41012 Seville, Spain

**Keywords:** deep learning, brainstem segmentation, medical imaging processing, brain MRI segmentation, convolutional neural networks, U-Net

## Abstract

**Highlights:**

**What are the main findings?**

- Superior Segmentation Performance: The proposed modified U-Net architecture (with attention-enhanced skip connections and inception modules) significantly outperforms three comparative approaches in brainstem parcellation, achieving higher scores across all substructures (medulla, pons, and mesencephalon) and the whole brainstem.
- Volume Differences Across Groups: Automated segmentation reveals distinct volumetric patterns, with controls exhibiting larger volumes (whole brainstem: 1.62) compared to preclinical (1.49) and patient groups (1.12), suggesting potential atrophy linked to disease progression.

**What is the implication of the main finding?**

- Clinical Utility: The method’s accuracy and robustness support its potential for precise brainstem assessment in neurodegenerative disorders, enabling earlier detection of structural changes (e.g., reduced medulla volume in patients: 0.26 vs. 0.31 in controls).
- Technical Advancements: The success of attention mechanisms and inception modules highlights their value for complex anatomical segmentation, paving the way for similar adaptations in other small-structure parcellation tasks.

**Abstract:**

Spinocerebellar ataxia type 2 (SCA2) is a neurodegenerative disorder marked by progressive brainstem and cerebellar atrophy, leading to gait ataxia. Quantifying this atrophy in magnetic resonance imaging (MRI) is critical for tracking disease progression in both symptomatic patients and preclinical subjects. However, manual segmentation of brainstem subregions (mesencephalon, pons, and medulla) is time-consuming and prone to human error. This work presents an automated deep learning framework to assess brainstem atrophy in SCA2. Using T1-weighted MRI scans from patients, preclinical carriers, and healthy controls, a U-shaped convolutional neural network (CNN) was trained to segment brainstem subregions and quantify volume loss. The model achieved strong agreement with manual segmentations, significantly outperforming four U-Net-based benchmarks (mean Dice scores: whole brainstem 0.96 vs. 0.93–0.95, pons 0.96 vs. 0.91–0.94, mesencephalon 0.96 vs. 0.89–0.93, and medulla 0.95 vs. 0.91–0.93). Results revealed severe atrophy in preclinical and symptomatic cohorts, with pons volumes reduced by nearly 50% compared to controls (*p* < 0.001). The mesencephalon and medulla showed milder degeneration, underscoring regional vulnerability differences. This automated approach enables rapid, precise assessment of brainstem atrophy, advancing early diagnosis and monitoring in SCA2.

## 1. Introduction

Spinocerebellar ataxia type 2 (SCA2) is a rare neurodegenerative disorder characterized by progressive degeneration of the brainstem and cerebellum. As one of the most prevalent spinocerebellar ataxias globally [1,2,3,4], it exhibits a notably high incidence in Holguin, Cuba [1,5]. Clinical manifestations include a cerebellar syndrome, slowing of the saccadic ocular movements, cognitive disorders, sensory neuropathy, etc. [6].

Three patterns of macroscopic atrophy reflecting damage of different neuronal systems are recognized in spinocerebellar ataxias, named spinal atrophy (SA), olivopontocerebellar atrophy (OPCA), and cortico-cerebellar atrophy (CCA) [7]. Neuroimaging plays a pivotal role in diagnosing neurodegenerative disorders, including modalities like magnetic resonance imaging (MRI), single-photon emission computed tomography (SPECT), and positron emission tomography (PET) [8]. Due to its anatomical nature, MRI remains the gold standard for structural segmentation and volumetrics, allowing visualization of SA, OPCA, and CCA [9]. According to recent literature [10,11], MRI is one of the most common biomarker candidates for spinocerebellar ataxias.

Brainstem atrophy has been documented across both symptomatic and prodromal stages of SCA2 [1,2,3,12,13,14,15,16,17,18,19,20]. However, most studies rely on manual segmentation, a method constrained by time-intensive workflows, inter-rater variability, and scalability limitations in large cohorts. To address these challenges, this work introduces an automated deep learning framework for quantifying volumetric changes in SCA2 patients, preclinical carriers, and healthy controls.

Convolutional neural networks (CNNs) have achieved state-of-the-art performance across diverse domains, including handwritten digit classification [21], face and contour detection [22], and automatic video processing [23,24]. In neuroscience, the applications of CNNs include the classification of electrooculograms and electroencephalograms [25,26], neurological behavior analysis and prediction [27,28,29], ataxic gait monitoring and classification [30,31,32,33,34], and speech recognition and processing for neurodegenerative diseases [35,36]. However, the atrophy estimation requires structural, anatomical data (e.g., structural MRI).

In neuroimaging, CNNs have become indispensable for brain lesion segmentation [37], structural parcellation [38,39,40], and neurological disease classification [41]. The most common architectures include U-Net [42,43,44,45], ResNet [46], and VGG-Net [47], leveraging adversarial training [48] and hierarchical feature extraction to enhance robustness. Some important advances in brain structure segmentation include the 3D cerebellum parcellation used by Han et al. [38], highlighting their potential for fine-grained neuroanatomical analysis, and the 2D approach proposed by Faber et al. [44] and Morell-Ortega et al. [39]. Two-dimensional convolutional models exhibit greater computational efficiency and lower resource demands by processing individual image slices independently. However, 3D convolutional models tend to achieve superior segmentation performance by leveraging volumetric spatial context, which is critical for accurately analyzing anatomical continuity and pathological structures across adjacent slices in neuroimaging data.

Recent architectural innovations have significantly advanced CNN-based segmentation performance in neuroimaging through sophisticated feature refinement and multi-scale processing. Convolutional Block Attention Modules (CBAM) [49,50,51] enhance segmentation precision by sequentially applying channel and spatial attention mechanisms, enabling the model to focus on diagnostically relevant features while suppressing noise. This approach has been particularly valuable for heterogeneous tumor regions and subtle subcortical boundaries. Another key component is the inception module [52,53,54,55], which addresses scale variance through parallel convolutional pathways with differing receptive fields, capturing both local texture details and global anatomical context essential for brain structures. The adoption of self-attention for brain structures and lesion segmentation [56,57] has introduced a powerful mechanism for modeling long-range contextual dependencies by computing interactions between all pairs of positions in a feature map. It can directly capture complex, non-local relationships, which are often challenging for local convolutions to grasp. However, this global receptive field comes at a prohibitive computational cost, with memory and time complexity rapidly increasing with spatial resolution, making it often impractical for high-resolution 3D medical volumes.

Building on prior work by Cabeza-Ruiz et al. [54], this study applies CNNs to brainstem segmentation in MRI, with a focus on mesencephalon, pons, and medulla volumetric changes. The main advancement over the previous architecture is the incorporation of CBAM, which greatly improves the model’s accuracy and allows an accurate segmentation of brainstem structures while maintaining a small number of parameters. To date, no studies have employed deep learning to compare brainstem atrophy on SCA2 in Cuba. This approach aims to establish a scalable, objective tool for identifying early biomarkers of SCA2 progression.

## 2. Materials and Methods

The proposed model architecture builds upon the method by Cabeza-Ruiz et al. [54], which uses a 3D U-Net-like framework to perform volumetric segmentations. This design processes the input images in their native 3D spatial context, preserving important anatomical relationships. The core symmetric encoder–decoder structure employs four downsampling and four corresponding upsampling operations. To enhance feature extraction, each convolutional layer was replaced with a modified inception module [58]. Our adaptation of the inception module (IM) simplifies the original design by reducing the four parallel paths to two core branches. The main branch processes data through a sequence of three convolutional layers with kernel sizes of (1 × 1 × 1), (3 × 3 × 3), and (3 × 3 × 3), with the feature maps from each step preserved for later concatenation. Concurrently, a secondary branch processes the same input through a max-pool layer and a convolutional layer with a (1 × 1 × 1) kernel. The outputs of these two branches are then concatenated, and the resulting feature map is normalized using instance normalization. This hierarchical structure facilitates efficient processing of multi-scale features while maintaining low memory requirements. The inclusion of (1 × 1 × 1) convolutions within the IM optimizes the trade-off between computational efficiency and multi-scale feature representation by dimensionality reduction, a common strategy in modern CNN architectures.

Additionally, skip connections were refined using three consecutive Convolutional Block Attention Modules (CBAM) [59]. This modification enables the model to focus on spatially and channel-wise relevant features in skip connections. The CBAM operates through two sequential sub-modules: a Channel Attention Module (CAM) followed by a Spatial Attention Module (SAM). The CAM first applies simultaneous global max-pooling and average-pooling to the input features. The resulting vectors are separately processed by a shared multi-layer perceptron, then summed, and passed through a sigmoid activation function to generate a channel attention vector. This vector is multiplied with the original input features. The output is then passed to the SAM, which applies channel-wise max-pooling and average-pooling, concatenates the results, and processes them through a convolutional layer with a (7 × 7 × 7) kernel and a sigmoid activation to produce a spatial attention map. For this research, the original convolutional layer with a (7 × 7 × 7) kernel was replaced by three consecutive convolutions with a kernel size of (3 × 3 × 3), allowing us to keep the same receptive field while using fewer parameters. The output map of the SAM is multiplied with the features from the CAM to yield the final weighted output.

**Figure 1:**
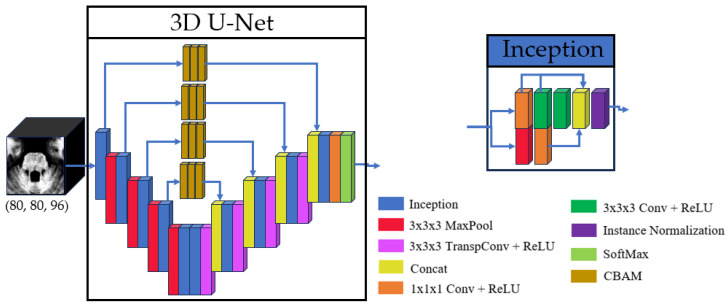
Basic structure of the 3D U-Net and the inception used.

**Figure 2:**
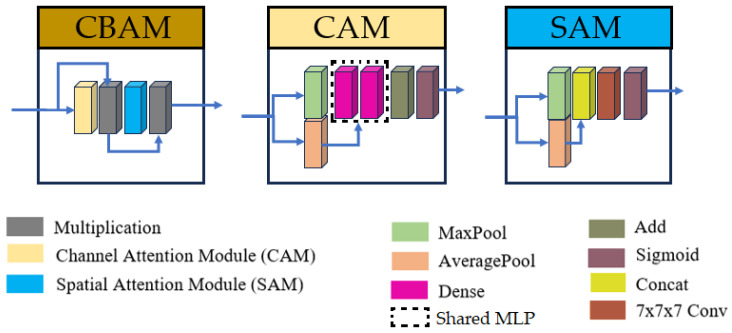
Structures of Convolutional Block Attention Module (CBAM), Channel Attention Module (CAM), and Spatial Attention Module (SAM). For the current research, the convolution with kernel size (7 × 7 × 7) was replaced by three consecutive convolutions with kernel sizes (3 × 3 × 3).

Given hardware limitations, the model was designed to balance computational efficiency with performance, ensuring feasibility on available infrastructure while maintaining robust segmentation accuracy. The inclusion of inception modules and CBAM in the proposed U-Net variant was motivated by the need to address two key challenges in brainstem segmentation: (1) the multi-scale nature of anatomical features and (2) the subtle intensity changes between adjacent substructures. Inception modules enable efficient multi-scale feature extraction, while the stacked CBAM refine the skip connections to prioritize anatomically relevant regions. The overall U-Net model and inception architectures are illustrated in Figure 1, with detailed schematics of the CBAM provided in Figure 2.

This study employed a cohort of 42 MRI scans obtained from the Cuban Neurosciences Center. These scans correspond to 25 individuals, comprising five healthy controls, seven preclinical subjects, and 13 SCA2 patients. The participants belong to different ages, SARA scores, and years of SCA2 evolution (see Table 1). This study was conducted in accordance with the Declaration of Helsinki and approved by the Research Ethics Committee of the Cuban Center for Neuroscience in November 2020. Written informed consent was obtained from all subjects involved in this study. MRI data were acquired using a Siemens 3T Allegra (Siemens Medical Solutions, Erlangen, Germany) system equipped with an 8Ch TxRx Head coil and running Syngo MR VA35A software. A high-resolution T1-weighted MPRAGE sequence was used to acquire anatomical images with the following parameters: TR = 2400 ms, TE = 2.36 ms, TI = 1000 ms, flip angle = 8°, and slice thickness = 0.8 mm.

### 2.1. Image Preparation

**Figure 3:**
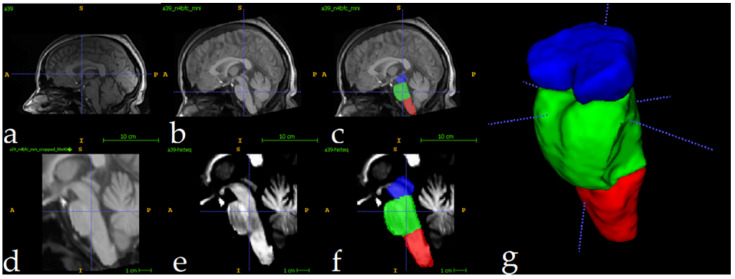
Full preprocessing routine for a single image. Original image (**a**), followed by N4 Norm.+MNI Registration (**b**) and manual label superposition (**c**). Follows the result of crop operation (**d**) and intensity normalization (**e**). Image (**f**) shows the manually segmented labels in the cropped region, and (**g**) shows a 3D view. Label colors: medulla (red), pons (green), and mesencephalon (blue).

The full preparation process for one single image can be depicted in Figure 3. All MRI scans underwent preprocessing to ensure consistency and improve segmentation accuracy. First, N4 bias field correction [60] was applied to address intensity inhomogeneities, enhancing image quality for subsequent analysis. Following this, each scan was registered to the ICBM 2009c nonlinear symmetric template [61] using Advanced Normalization Tools (ANTS) [62]. For the registration process, an affine initialization was calculated to find a good initialization for further refinement, using a search factor of 15. Then, a three-stage approach was followed: rigid (affine iterations: [1500, 1000, 500, 100], using the full mask of the MNI template), affine (affine iterations: [1500, 1000, 500, 100], using the brain mask of the MNI template), and symmetric normalization (SyNOnly) with default parameters.

**Figure A1:**
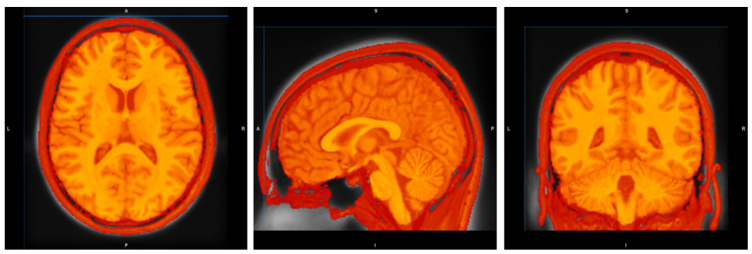
Registration of a sample image of the cohort. From left to right: axial, sagittal, and coronal views.

The ICBM 2009c template was selected for its validated ability to represent adult brain anatomy and compatibility with other neuroimaging studies [63,64,65]. The described hierarchical registration pipeline minimizes anatomical mismatch by progressively aligning scans to the template’s symmetric space. Figure 3a shows the original image, and Figure 3b displays the result of N4+MNI registration. To quantitatively evaluate the effectiveness of the registration phase, we computed the Dice Similarity Coefficient (DSC) between the brainstem mask of each registered subject MRI and a predefined brainstem mask of the MNI template. The analysis yielded a mean DSC of 0.92 ± 0.01, indicating excellent alignment across the cohort. An example of this registration is provided in Figure A1.

To optimize computational efficiency, MRI scans were cropped to focus exclusively on the brainstem region. Using the segmentations of the training set as reference, a standardized region of interest (ROI) measuring 80 × 80 × 96 voxels was extracted for each scan. This approach greatly reduced the computational load, decreasing processed volumes from approximately 8.5 million voxels (per full scan) to 614,400 voxels. The cropped ROIs enabled efficient model training and inference while preserving relevant anatomical data for brainstem analysis. Figure 3d shows the result of the cropping operation. Following the crop, intensity normalization was applied for every image (Figure 3e). These volumes were used as inputs to the 3D U-Net. Figure 3f shows one fully preprocessed image overlapping with its manual segmentations.

### 2.2. Analysis Description

This study was implemented in Python 3.9, using TensorFlow [66] and Keras [67] for model development and training. The model was trained over 250 epochs using the Adam optimizer [68] with default parameters and a constant learning rate of 10^−4^. To mitigate overfitting, a dropout rate of 0.2 was applied before the final convolutional layer. The loss function used was one minus the average Dice score (DSC) across all channels; the DSC is computed as mentioned by Han et al. [38].

The experiment was conducted on a computer provided with an Intel Core i5-10500H microprocessor (Intel Corporation, Santa Clara, CA, USA), 16 GB RAM, and an NVIDIA RTX 3060 6 GB GPU (NVIDIA, Santa Clara, CA, USA) using mixed precision. The dataset was partitioned into 17 images for training, 3 for validation, and 22 for testing. Careful stratification ensured representation of all clinical categories (patients, controls, and preclinical carriers) in each subset, with the validation set containing the minimal case of one scan per category. Subject assignment followed a randomized distribution with no additional constraints. The final number of images per group in the partitions was: train (eight patients, seven preclinical, and two controls), validation (one per group), and test (fourteen patients, six preclinical, and two controls). To enhance model generalization, and given the small size of the available cohort, data augmentation techniques were applied to every image in the training set. The augmentation included random rotations (−15° to 15°), translations (±15 pixels in each direction), and flipping (probabilistic). For each image in the train/validation sets, 40 augmented images were generated. The final training set therefore consisted of 697 MRIs (17 original and 680 augmented), and the validation set contained 123 MRIs (3 original and 120 augmented). No augmentation was applied to the images of the test nor validation sets.

The proposed method was evaluated against four U-Net-based approaches: (1) an upscaled version of the model architecture by Cabeza-Ruiz et al. [54]; (2) the cerebellar parcellation network by Han et al. [38]; (3) the brainstem parcellation model by Magnusson et al. [69]; and (4) the whole-brain parcellation model used by Nishimaki et al. [40]. To ensure a fair and accurate comparison, a rigorous approach was employed for the implementation of the benchmark models. Wherever possible, the authors’ original code was used, as was the case for the model by Han et al. [38] and the previous architecture from Cabeza-Ruiz et al. [42]. For the method by Nishimaki et al. [56], a faithful translation of the official Torch code into the TensorFlow environment was performed. For the approach by Magnusson et al. [55], whose code was not available, the network was implemented based on the architectural description provided in their original paper.

To ensure a fair comparison, all models were trained under identical conditions, maintaining consistent training protocols (loss function, optimizer parameters, regularization strategies, number of epochs, and learning rate). Due to computational constraints, the model proposed by Nishimaki et al. [40] was impossible to train with the original filter sizes for each U-Net stage. To overcome this limitation, the number of filters was changed from [64, 128, 256, 512, 1024] to [32, 64, 128, 256, 512], ensuring a proper fit in our graphics card. The parcellating model by Han et al. [38] was reduced too, removing one of the encoder steps. To mitigate the possible negative effects of this reduction, the numbers of the filters in each encoder step were increased [48, 48, 96, 195, 384, 768] to [64, 64, 128, 256, 512]. Table 2 shows the number of filters used for each model, as well as the total number of parameters per approach.

The segmentations generated by the top-performing model were subsequently utilized for volumetric analyses, with regional volumes normalized as a percentage of total intracranial volume (%TICV). Additionally, we investigated potential clinical and developmental correlations by examining the relationship between brainstem subregion volumes, Scale for the Assessment and Rating of Ataxia (SARA) scores, disease duration, and CAG repeat length. These analyses aimed to elucidate whether volumetric variations in the medulla, pons, and mesencephalon were associated with the neurological detriment related to SCA2. The correlation *p*-values were adjusted via Bonferroni correction (12 tests).

### 2.3. Ablation Study: Quantifying the Contribution of CBAM

To quantitatively evaluate the contribution of the Convolutional Block Attention Modules (CBAM), an ablation study was conducted. A version of the proposed architecture was trained without the CBAM components on the skip connections. This ablated model demonstrated significantly hampered learning capabilities, with its DSC on the validation and training sets plateauing below 0.84 throughout 60 training epochs. In contrast, the full model incorporating CBAM exhibited a rapid improvement in performance, with the DSC escalating to 0.87 early in training and continuing its upward trajectory. This performance gap underscores the critical role of attention mechanisms, stabilizing the training process and enabling the model to achieve high-precision segmentation by effectively focusing on anatomically relevant features.

## 3. Results

The evaluation results demonstrate high segmentation accuracy across all regions of interest. Mean DSC exceeded 0.95 for all structures, with the highest score (0.97) achieved for the whole brainstem. The mesencephalon exhibited the lowest mean DSC (0.93), indicating consistent yet slightly reduced performance in this region. These results highlight the model’s robustness and reliability in segmenting brainstem subregions. Table 3 shows the mean DSC for each model evaluated in the test set. The evaluations include all the brainstem regions (medulla, pons, and mesencephalon) and the full brainstem. Table 4 shows the results of the Intersection over Union (IoU), Hausdorff Distance (HD95), Specificity, Sensitivity, and Precision, calculated for the full brainstem.

**Figure 4:**
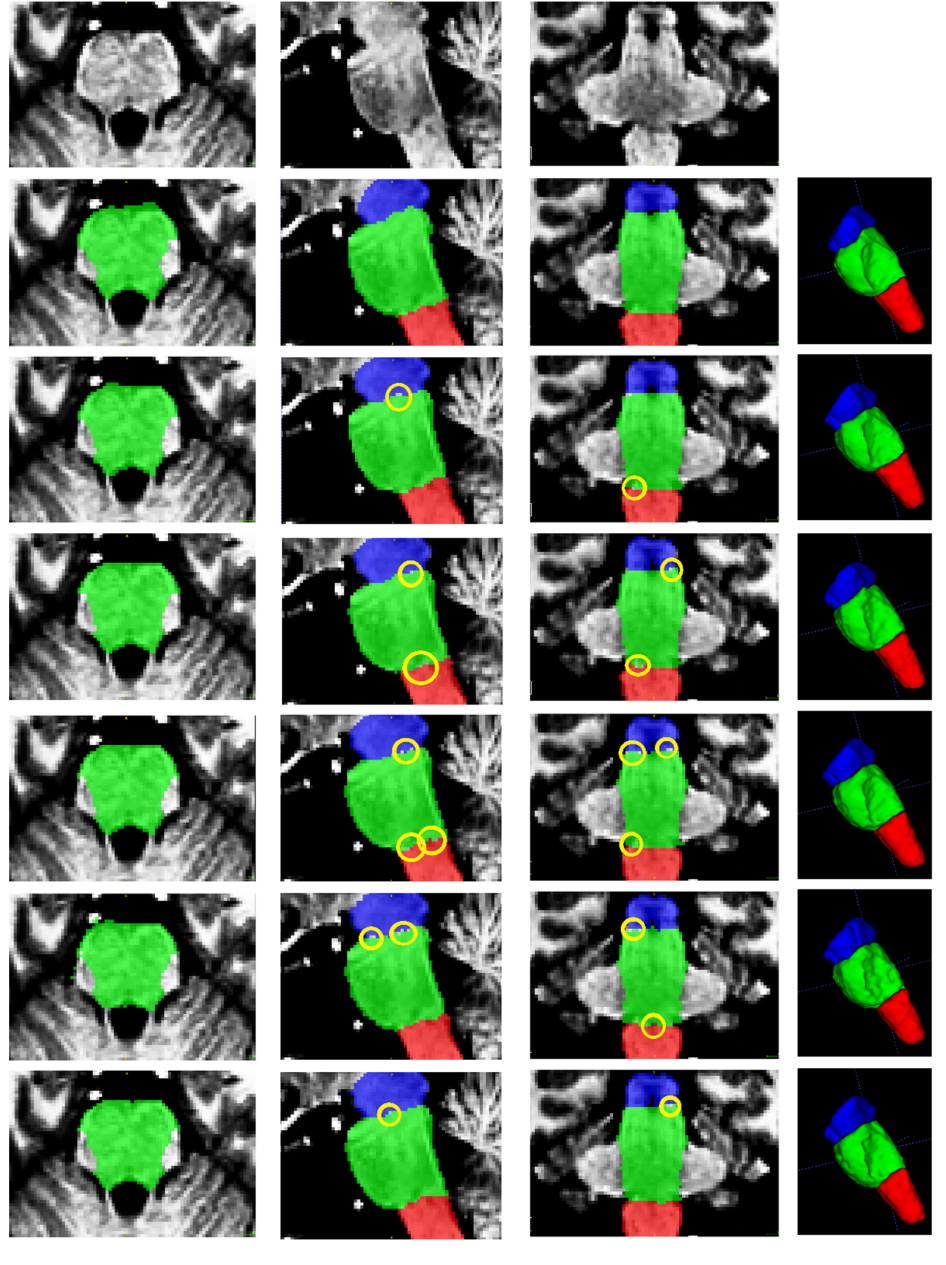
From top to bottom: original image, ground truth segmentation, and segmentations produced by this research, Cabeza-Ruiz et al. [54], Han et al. [38] (modified), Magnusson et al. [69], and Nishimaki et al. [40] (modified)**.** From left to right: axial, sagittal, coronal, and 3D views. Labels shown: medulla (red), pons (green), and mesencephalon (blue). The yellow ellipses indicate segmentation errors producing holes in the intersections of adjacent labels.

**Figure 5:**
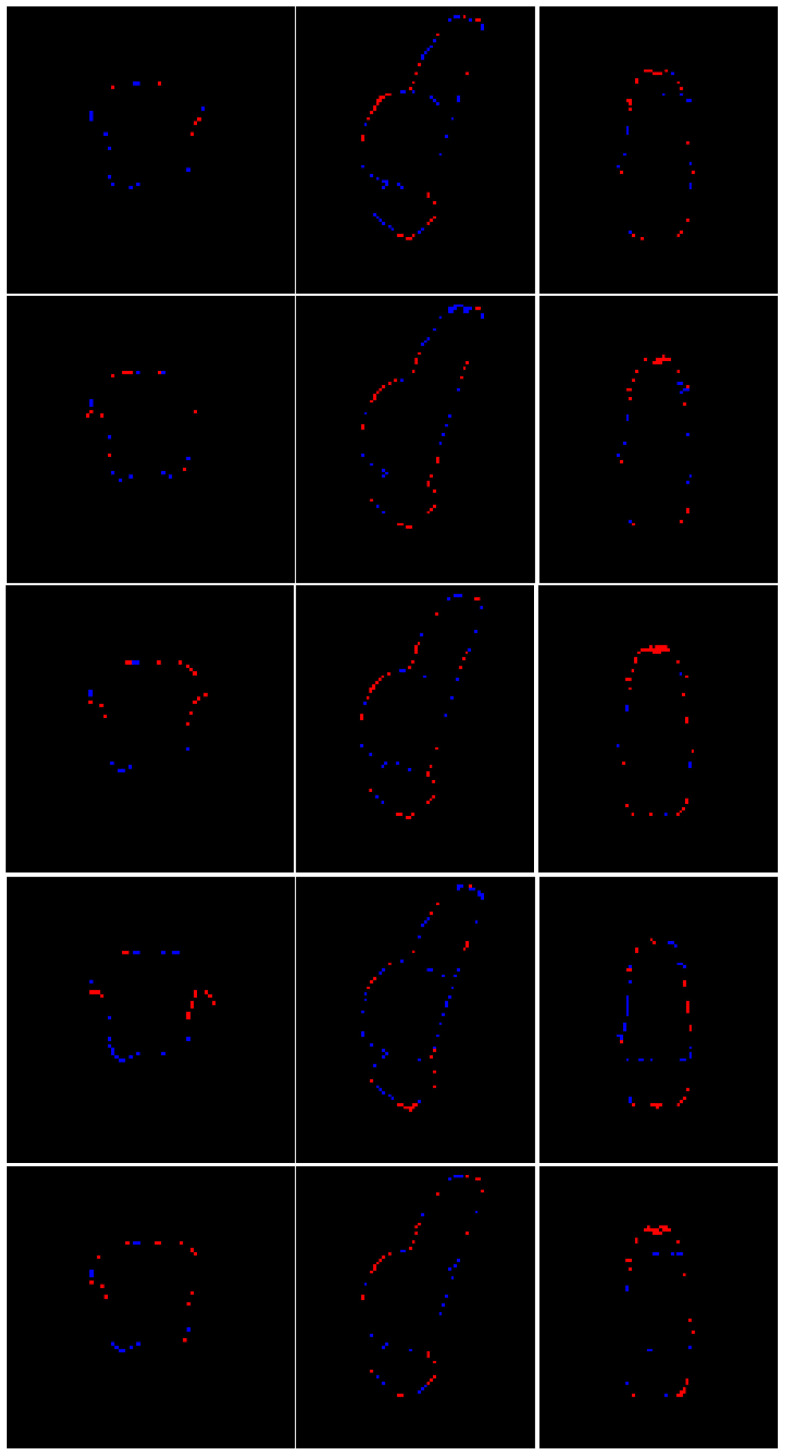
Multiplanar errors for a representative case. Blue: false negatives; red: false positives. From top to bottom: multiplanar errors for the segmentation produced by this research, Cabeza-Ruiz et al. [54], Han et al. [38] (modified), Magnusson et al. [69], and Nishimaki et al. [40] (modified)**.** From left to right: axial and sagittal views.

Figure 4 presents a qualitative comparison of segmentation results for a single case. The visual assessment of the segmentations generated by the five models reveals a consistent pattern in the spatial distribution of errors. In some cases, the models do not find the precise borders between adjacent labels, leaving small holes in segmentations (marked in yellow in Figure 4). The modification of the model by Nishimaki et al. [40] and the model by Magnusson et al. [69] also show irregular borders in the pons, producing artifacts in the 3D view. All models achieve high performance metrics (including DSC, HD95, Sensitivity, Specificity and Precision). Nevertheless, qualitative inspection of the multiplanar error maps (Figure 5) indicates that discrepancies predominantly occur along the outer boundaries of the brainstem rather than at the internal interfaces between substructures.

**Figure 6:**
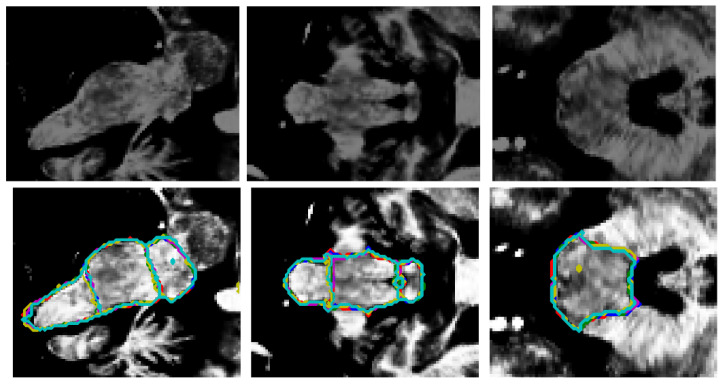
Original image ((**top**) row) and the superposition of the segmentation borders by all the models ((**bottom**) row). From left to right: sagittal, coronal, and axial views. Red: ground truth borders, blue: this research, green: Cabeza-Ruiz et al. [54], magenta: Han et al. [38] (modified), yellow: Magnusson et al. [69], cyan: Nishimaki et al. [40] (modified). The superposition of the lines indicates an overall good recognition of borders. The model by Magnusson et al. [69] (yellow) produced an artifact (axial view), creating a small hole in the segmentation.

**Figure 7:**
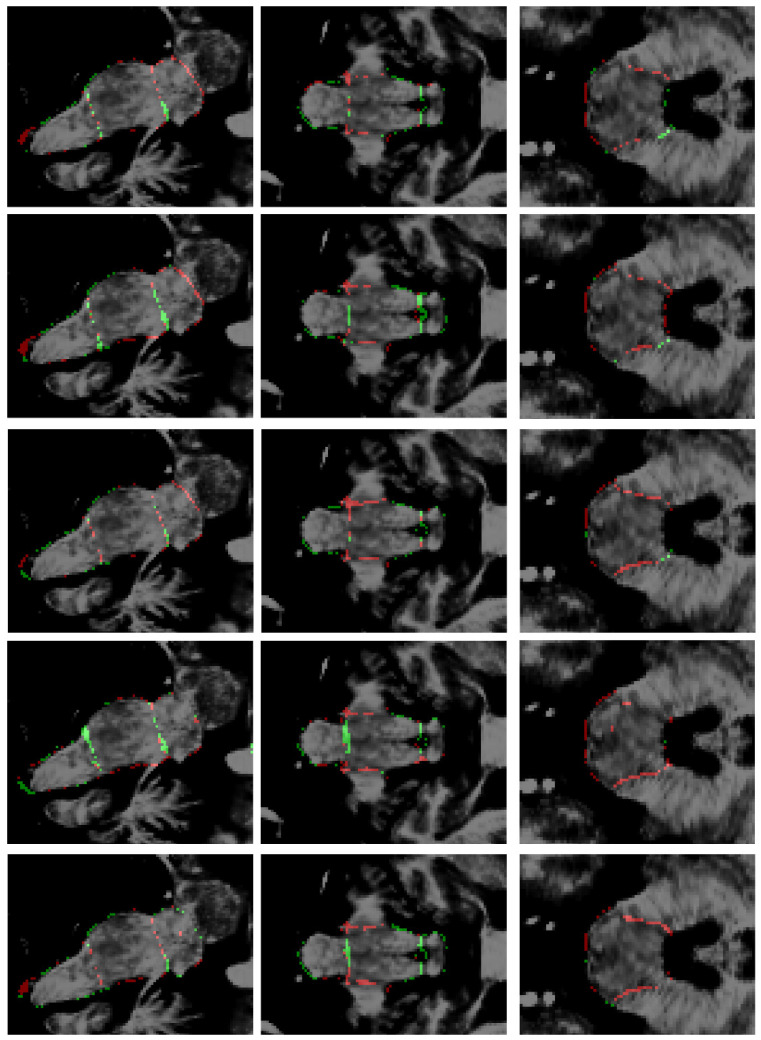
Errors in segmentations. From top to bottom: this research, Cabeza-Ruiz et al. [54], Han et al. [38] (modified), Magnusson et al. [69], and Nishimaki et al. [40] (modified). From left to right: sagittal, coronal, and axial views. Green: false positives; red: false negatives.

Figure 6 shows a superposition of the segmentations produced by all the models on another test image. As the image depicts, all the external borders are very similar, which means an overall good segmentation for all the models. An artifact produced by the model of Magnusson et al. [69] formed a small hole in the segmentation. Figure 7 presents the respective planar errors for each model on that same image. It may be appreciated that our model and the modification of Han et al. [38] did a better job identifying the correct borders between the adjacent brainstem regions.

As depicted in Figure 5 and Figure 7, the most common segmentation errors are placed in the outer boundary of the brainstem. This observation suggests that the models excel in delineating the internal architecture of the brainstem, accurately capturing the transitions between adjacent subregions, but exhibit minor inaccuracies in defining the precise exterior margins of the brainstem itself. The concentration of errors along the periphery may reflect inherent challenges in boundary definition due to partial volume effects at the brainstem’s interface with surrounding cerebrospinal fluid or adjacent tissues. Additionally, slight variations in image contrast or resolution near the edges could contribute to this phenomenon. Importantly, the robustness of internal parcellation underscores the ability of the models to learn and reproduce the complex anatomical relationships between substructures, which is critical for clinical and research applications. Future work could explore postprocessing refinements or targeted training strategies to further improve boundary precision without compromising the already high accuracy of internal segmentation.

Based on the information provided in Table 2, Table 3 and Table 4 and Figure 4, Figure 5, Figure 6 and Figure 7, the proposed method achieves superior performance while maintaining the lowest computational footprint. The model demonstrates comprehensive outperformance over competing methods. While all models achieve high DSC, our method consistently delivers the highest scores for the majority of structures, particularly the full brainstem. More importantly, the analysis of boundary-specific metrics (ASD and NSD) reveals that our segmentations are not merely volumetrically accurate but are also precisely aligned with the true anatomical boundaries. Our best-in-class ASD and NSD scores confirm superior surface accuracy. The HD95 of 2.71 mm indicates precise boundary delineation, comparable to Nishimaki et al.’s [40] marginally better 2.65 mm, but using a substantially smaller model.

Beyond accuracy, the proposed model stands out for its efficiency. With only 5.25 million parameters, it is significantly leaner than competing models, which range from 8.79 million to 22.6 million parameters. This efficiency does not come at the cost of performance, as the model matches or exceeds the scores in secondary metrics such as sensitivity (0.949) and precision (0.972). In contrast, larger models like Han et al. [38] and Nishimaki et al. [40] (with 21.64 M and 22.6 M parameters after modification, respectively) offer negligible improvements while demanding far greater computational resources. This makes the proposed approach particularly suitable for real-world clinical settings, where hardware limitations and inference speed are practical concerns.

The implications of these findings are substantial for both researchers and practitioners. For clinicians, the model’s high accuracy ensures reliable segmentation for diagnostic purposes. For researchers, its parameter efficiency translates to faster inference times and lower hardware costs, facilitating the deployment in resource-constrained environments. From a research perspective, this work establishes a new benchmark for balancing performance and efficiency in medical imaging segmentation. The improvements can be attributed to the two key architectural changes made to the U-Net: (1) the integration of attention mechanisms within skip connections to refine feature aggregation and (2) the replacement of conventional convolutional layers with modified inception modules to capture multi-scale contextual information more effectively. While future studies will explore further optimizations, the current model demonstrates that state-of-the-art accuracy does not come at the expense of larger models.

Quantitative evaluation of computational efficiency revealed segmentation times of <1 s per image on a GPU environment (NVIDIA RTX 3060 MOBILE, 6 GB GDDR6), while CPU-based processing (Intel Core i3-8145U, 8 GB DDR4 RAM) required 7 ± 0.89 s per case. These times represent great speed improvement compared to manual segmentation protocols while maintaining diagnostic-grade accuracy.

Using the segmentation results for all the images of the initial cohort, volumetric changes were calculated for SCA2 patients, preclinical subjects, and healthy controls. For this, all the segmentations were uncropped to the original dimensions of the template and then back-registered to the original space of the MRIs. Volumes were normalized as a percentage of the total intracranial volume (% TICV). The TICV was computed using ROBEX [70]. Consistent with prior findings by Reetz et al. [71], the comparison (Table 5) revealed a progressive volumetric reduction: SCA2 patients exhibited significantly smaller brainstem subregion volumes compared to preclinical subjects, which in turn were reduced relative to healthy controls. These findings validate the model’s ability to detect subtle neuroanatomical changes, reinforcing its utility in clinical assessment of neurodegenerative disorders.

The most pronounced differences were observed in the pons, with mean volumes of 0.47% TICV for patients, 0.76% TICV for preclinical subjects, and 0.82% TICV for controls. Notably, the median volume for controls was nearly double that of patients. Differences between preclinical subjects and controls were less pronounced. In the mesencephalon, mean volumes were 0.40% TICV for patients, 0.44% TICV for preclinical subjects, and 0.48% TICV for controls. The medulla exhibited the smallest volumetric differences, with values of 0.26%, 0.29%, and 0.31% TICV for patients, preclinical subjects, and controls, respectively. At the whole brainstem level, mean volumes were 1.12%, 1.49%, and 1.62% TICV for patients, preclinical subjects, and controls, respectively. This highlights the progressive nature of brainstem atrophy in SCA2.

The automated segmentations were also used to assess the relationship between brainstem subregion volumes and clinical measures, including the SARA scores, disease duration, and CAG repeat length (Table 6). The analysis included eleven SCA2 patients and eight preclinical carriers (one patient was excluded due to missing data). For disease duration, correlations were restricted to the patient cohort. All the brainstem subdivisions showed significant negative correlations with SARA scores (pons: r = −0.69, *p* < 0.01; whole brainstem: r = −0.71, *p* < 0.01), indicating that smaller volumes are associated with worse ataxia severity. Notably, among the three substructures, the pons demonstrated the strongest association, aligning with its known role in motor coordination. Disease duration and CAG repeat length did not correlate prominently with any of the structures, as after Bonferroni corrections all *p*-values were greater than 0.05.

## 4. Discussion

This study presented a deep learning-based framework for the quantification of volumetric changes in the brainstem of SCA2 patients and preclinical subjects compared to healthy controls. To the best of our knowledge, this represents the first such study conducted in Cuba, addressing a critical need for accessible and efficient tools to study neurodegenerative diseases in resource-constrained settings.

The success of the approach stems from the inherent advantages of the 3D U-Net for medical image segmentation. Unlike classical techniques (e.g., atlas-based or graph-cut methods) that rely on handcrafted features (which often fail to capture complex anatomical variability [72]), CNNs automatically learn discriminative hierarchical features, enabling precise parcellation of challenging structures [73]. The proposed 3D U-Net architecture incorporates two key changes: stacked attention modules in skip connections and modified inception modules replacing standard convolutions. The attention modules enable precise localization of anatomical boundaries by selectively emphasizing relevant spatial features. On the other side, the inception modules improve the U-Net’s capability to capture multi-scale contextual information critical for distinguishing between adjacent brainstem subregions. This advanced and complex architecture achieves expert-level segmentation accuracy, with DSC > 0.95 for all brainstem substructures.

The results demonstrate that deep learning techniques can effectively characterize brainstem atrophy on SCA2, enabling rapid differentiation between patients, preclinical subjects, and controls. The findings demonstrate a robust inverse relationship between pons volume and SARA scores (r = −0.69, *p* < 0.01), underscoring the pons’ pivotal role in SCA2-related motor dysfunction. This correlation suggests that automated brainstem volumetry serves as a biomarker for clinical trials, enabling early intervention in preclinical carriers. Future longitudinal studies will validate these associations and explore multimodal imaging to refine prognostic models. These findings suggest that the proposed framework can be integrated into larger neuroimaging pipelines to assess volumetric changes in SCA2 patients and preclinical subjects. The development of user-friendly software based on this approach could provide clinicians with powerful tools for rapid diagnostics, helping to evaluate disease progression. The use of such tools could improve the success of patient care and support early intervention strategies.

The computational efficiency of the proposed method offers significant advantages for clinical implementation. The brainstem-specific segmentation is completed in under one second per image on a GPU, representing a substantial reduction in time compared to manual segmentation. Even when processed on a CPU, a segmentation time of approximately seven seconds per case remains highly efficient for clinical settings. It is important to note that these times reflect the brainstem-specific segmentation only, with the full pipeline (including registration) requiring additional computation. The choice between GPU and CPU implementation presents practical considerations: GPU acceleration enables real-time processing for clinical workflows, while CPU processing remains viable for resource-constrained environments at the cost of increased processing time. Importantly, both approaches maintain equivalent segmentation accuracy, with the computational differences arising solely from hardware capabilities. For large-scale deployments, GPU implementations are recommended when available, as they provide the most balanced combination of speed and precision. The method’s memory requirements make it deployable on most modern medical imaging workstations without specialized hardware.

### Limitations and Future Work

The main limitations of the current work can be summarized as follows:

(a) Registration dependency: While the hierarchical registration pipeline ensures robust alignment to the ICBM 2009c template, this preprocessing step might introduce critical limitations. A failure in the registration step will inevitably lead to a wrong segmentation. In addition, the ICBM 2009c template may not generalize to other populations, potentially biasing volumetric estimates.
(b) Regional bias due to dataset homogeneity: All the collected data belongs to Cuban individuals, which may limit the generalizability to global SCA2 populations with differing genetic/environmental profiles.
(c) Small cohort size: While the proposed model demonstrates strong performance in the used cohort, deep learning models typically benefit from larger and more diverse datasets to ensure robustness across populations and imaging protocols.
(d) Cross-sectional design: Volumetric differences are reported at a single timepoint, precluding causal inferences about atrophy progression.
(e) Resource-constrained experiments: Due to resource constraints, the models proposed by Nishimaki et al. [40] and Han et al. [38] had to be reduced to 22.6 M and 21.6 M parameters, respectively, which surely affected the quality of the segmentations.

To assess these limitations, future research will be mainly oriented to the development of registration-free pipelines, allowing for enhanced robustness and generalizability. The use of vision transformers could be a possible path for exploration, but their innate need for computational resources might negatively impact the processing time. Another way to avoid registration could be using a cascaded approach, similar to that proposed by Han et al. [38]. In addition, increasing the size of the dataset will positively influence the model’s generalizability. For this purpose, new SCA2 patients and preclinical carriers will be added to the cohort. The extension of the dataset with patients suffering other neurological diseases (i.e., other types of SCA and Parkinson’s Disease) will be assessed in future research too, as a way to increase robustness and generalization. Multimodal imaging will be explored too, taking advantage of the information extracted from multiple modalities (DTI, fMRI, and T2-weighted MRI) to map microstructural degeneration. Furthermore, the use of longitudinal MRI data will also be explored, aiming for a more intrinsic correlation between volumetric trajectories and clinical decline. Finally, future work must validate findings on high-end hardware to isolate architecture-vs.-parameter effects.

## 5. Conclusions

This study introduced a deep learning-based framework to quantify brainstem atrophy in SCA2 patients, preclinical subjects, and healthy controls, representing a pioneering effort in Cuba. By achieving a mean DSC above 0.96 for the whole brainstem and 0.95 for its subregions, the approach demonstrates high accurateness in detecting significant volumetric differences, particularly in the pons. The experiment showed highly negative correlations between all brainstem structures and SARA scores. These findings highlight the potential of deep learning to address critical gaps in neuroimaging analysis. The method used enables rapid, scalable assessments, reducing reliance on time-intensive manual segmentations and supporting earlier diagnosis of SCA2. While the framework demonstrates high accuracy, its reliance on registration and homogeneous data limits immediate clinical translation. Future work will prioritize registration-free architectures, multi-center validation, and longitudinal designs to establish causal links between atrophy and symptom progression. By addressing these limitations, we aim to deploy this tool as a scalable solution for neurodegenerative disease monitoring.

## Figures and Tables

**Table 1:** Demographic information of the cohort’s individuals.

Data	Value Range
Age (years)	25 to 72
SARA score (patients and preclinical)	0.0 to 39
CAG repeat	36 to 40
Evolution years (patients)	1 to 31

**Table 2:** For each model, the number of filters after each encoder step, and the total number of parameters.

Approach	Number of Output Filters in Encoder	Number of Parameters
This research	[32, 64, 128, 256, 512]	5,254,266
Cabeza-Ruiz et al. [54]	[64, 128, 256, 512, 512]	8,788,376
Han et al. [38] (modified)	[64, 64, 128, 256, 512]	21,641,792
Magnusson et al. [69]	[32, 64, 128, 256, 512]	10,796,252
Nishimaki et al. [40] (modified)	[32, 64, 128, 256, 512]	22,598,862

**Table 3:** Mean dice scores and stdev achieved for each model in the test set. The best scores have been bolded.

Label	Mean DSC ± stdev				
	This Research	Cabeza-Ruiz et al. [54]	Han et al. [38] (Modified)	Magnusson et al. [69]	Nishimaki et al. [40] (Modified)
Mesencephalon	**0.96** ± 0.022	0.92 ± 0.019	0.93 ± 0.019	0.89 ± 0.031	0.91 ± 0.022
Pons	**0.96** ± 0.015	0.94 ± 0.014	0.94 ± 0.013	0.91 ± 0.029	0.93 ± 0.014
Medulla	**0.95** ± 0.021	0.93 ± 0.020	0.92 ± 0.021	0.91 ± 0.023	0.93 ± 0.021
Full brainstem	**0.96** ± 0.008	0.95 ± 0.008	0.95 ± 0.007	0.93 ± 0.013	0.95 ± 0.007

**Table 4:** Mean Intersection over Union (IoU), Hausdorff Distance (HD95), Specificity, Sensitivity, Precision, Average Symmetric Surface Distance (ASD), and Normalized Surface Dice (NSD). Evaluations performed using the full brainstem. The best scores have been bolded.

Measures	Score (Mean Value)				
	This Research	Cabeza-Ruiz et al. [54]	Han et al. [38] (Modified)	Magnusson et al. [69]	Nishimaki et al. [40] (Modified)
IoU	**0.914** ± 0.01	0.904 ± 0.01	0.906 ± 0.01	0.886 ± 0.05	0.906 ± 0.01
HD95 (mm)	2.71	3.02	2.78	3.29	**2.65**
Specificity	0.998 ± 0.0007	0.998 ± 0.0007	0.998 ± 0.0006	0.997 ± 0.001	0.998 ± 0.0007
Sensitivity	**0.949** ± 0.01	0.938 ± 0.02	0.941 ± 0.02	0.928 ± 0.05	**0.947** ± 0.01
Precision	**0.972** ± 0.01	0.963 ± 0.01	0.950 ± 0.01	0.939 ± 0.01	0.953 ± 0.01
ASD	**0.052**	0.058	0.058	0.079	**0.054**
NSD	**0.993**	0.991	**0.992**	0.985	**0.992**

**Table 5:** Mean volumes for SCA2 patients, preclinical carriers, and control subjects. P: *p*-values from the Kruskal–Wallis test.

Brainstem Section	Mean Volumes (% TICV)			P
	Patients	Preclinical	Controls	
Mesencephalon	0.4	0.44	0.48	0.007
Pons	0.47	0.76	0.82	<0.0001
Medulla	0.26	0.29	0.31	0.00012
Whole brainstem	1.12	1.49	1.62	<0.0001

**Table 6:** Correlations of brainstem volumes (%TICV) with clinical measures: SARA scores and CAG repeats (patients + preclinical; *n* = 19 total); disease duration (patients only; *n* = 11). P: *p*-values adjusted for multiple comparisons via Bonferroni correction (α = 0.0042).

Brainstem Section/Score	SARA		Disease Duration		CAG Repeat	
	Corr	P	Corr	P	Corr	P
Mesencephalon	−0.62	<0.05	−0.58	0.72	−0.49	0.36
Pons	−0.69	<0.01	−0.35	1.00	−0.56	0.14
Medulla	−0.62	<0.05	−0.22	1.00	−0.43	0.72
Whole brainstem	−0.71	<0.01	−0.37	1.00	−0.55	0.15

## Data Availability

The preprocessed (totally anonymized) MRIs used in this study can be shared with authors upon a reasonable request. The source code for this study is publicly available at the GitHub repository: https://github.com/robbinc91/mipaim_unet (last accessed on 20 August 2025).

## Abbreviations

The following abbreviations are used in this manuscript:

SCA2	spinocerebellar ataxia type 2
MRI	magnetic resonance imaging
CNN(s)	convolutional neural network(s)
SA	spinal atrophy
OPCA	olivopontocerebellar atrophy
CCA	cortico-cerebellar atrophy
SPECT	single-photon emission computed tomography
PET	positron emission tomography
CBAM	Convolutional Block Attention Module
IM	inception module
CAM	Channel Attention Module
SAM	Spatial Attention Module
DSC	Dice Similarity Coefficient
ROI	region of interest
TICV	total intracranial volume
SARA	Scale for the Assessment and Rating of Ataxia
IoU	Intersection over Union
HD95	95th percentile Hausdorff Distance
ASD	Average Symmetric Surface Distance
NSD	Normalized Surface Dice

## Appendix A

Appendix A provides a visual demonstration of the image registration process, a critical preprocessing step for ensuring spatial normalization across all subjects in the cohort. The figure below illustrates the successful alignment of a representative subject’s T1-weighted MRI to the ICBM 2009c nonlinear symmetric template.
